# Mesenchymal Stromal Cells Promote Axonal Outgrowth Alone and Synergistically with Astrocytes via tPA

**DOI:** 10.1371/journal.pone.0168345

**Published:** 2016-12-13

**Authors:** Jian-Yong Qian, Michael Chopp, Zhongwu Liu

**Affiliations:** 1 Department of Neurology, Henry Ford Hospital, Detroit, Michigan, United States of America; 2 Department of Physics, Oakland University, Rochester, Michigan, United States of America; University of Louisville, UNITED STATES

## Abstract

We reported that mesenchymal stromal cells (MSCs) enhance neurological recovery from experimental stroke and increase tissue plasminogen activator (tPA) expression in astrocytes. Here, we investigate mechanisms by which tPA mediates MSC enhanced axonal outgrowth. Primary murine neurons and astrocytes were isolated from wild-type (WT) and tPA-knockout (KO) cortices of embryos. Mouse MSCs (WT) were purchased from Cognate Inc. Neurons (WT or KO) were seeded in soma side of Xona microfluidic chambers, and astrocytes (WT or KO) and/or MSCs in axon side. The chambers were cultured as usual (normoxia) or subjected to oxygen deprivation. Primary neurons (seeded in plates) were co-cultured with astrocytes and/or MSCs (in inserts) for Western blot. In chambers, WT axons grew significantly longer than KO axons and exogenous tPA enhanced axonal outgrowth. MSCs increased WT axonal outgrowth alone and synergistically with WT astrocytes at both normoxia and oxygen deprivation conditions. The synergistic effect was inhibited by U0126, an ERK inhibitor, and receptor associated protein (RAP), a low density lipoprotein receptor related protein 1 (LRP1) ligand antagonist. However, MSCs exerted neither individual nor synergistic effects on KO axonal outgrowth. Western blot showed that MSCs promoted astrocytic tPA expression and increased neuronal tPA alone and synergistically with astrocytes. Also, MSCs activated neuronal ERK alone and synergistically with astrocytes, which was inhibited by RAP. We conclude: (1) MSCs promote axonal outgrowth via neuronal tPA and synergistically with astrocytic tPA; (2) neuronal tPA is critical to observe the synergistic effect of MSC and astrocytes on axonal outgrowth; and (3) tPA mediates MSC treatment-induced axonal outgrowth through the LRP1 receptor and ERK.

## Introduction

Stroke is one of the leading causes of death and disability worldwide. Currently, one of a few evidence-based acute stroke treatments is thrombolysis induced by intravenous administration of recombinant tissue plasminogen activator (tPA). Unfortunately, only a small percentage of patients benefit from this treatment primarily due to a narrow therapeutic time window of 4.5 hours [1–3].

Restorative therapy for stroke may provide a complementary and an alternative therapeutic approach [4–7], and functional recovery is key to ameliorate post-stroke deficits and improve life quality of stroke patients [8]. Among potential restorative treatments, exogenous cell-based therapies have been extensively studied, and multipotent mesenchymal stromal cell (MSC) has emerged as a promising therapeutic candidate [9–13]. We and others reported that exogenous administration of MSCs after experimental stroke facilitates neurite outgrowth, accelerates axonal sprouting and regeneration, enhances intercortical and intracortical axonal connections and improves neurologic recovery after stroke [14–19]. In vivo data revealed that reactive astrocytes promote brain plasticity and recovery from stroke, and astrocytes are involved in MSC mediated neurological recovery [20, 21].

Astrocytes are a major constituent of the central nervous system, with versatile functions [22]. In the developing brain, astrocytes support and direct neurite extension through their synthesis of cell surface and extracellular matrix (ECM) molecules [13, 23]. In the adult animals after stroke, axons may also acquire their potential for outgrowth from neighboring astrocytes and help establish contacts with existing circuits in the CNS [24]. MSCs stimulate neurotrophins and growth factors, including vascular endothelial growth factor (VEGF) [25, 26], basic fibroblast growth factor (bFGF) [19, 27] and brain derived neurotrophic factor (BDNF) [28, 29] within reactive astrocytes in response to the ischemic brain environment [30, 31].

In response to MSC treatment, white matter changes are mediated by astrocytes via increased tPA activity [18, 32]. In vitro data suggest that the MSC induced activation of tPA in astrocytes promotes neurite outgrowth after ischemia [32, 33], and MSCs significantly increase tPA expression and concomitantly decrease PAI-1 expression in astrocytes [33]. Therefore, exogenously administered MSCs may promote neurite remodeling in the CNS via astrocytic tPA and thereby improve neurological recovery. Recently, Mantuano et al reported that in PC12 and N2a neuron-like cells, tPA binds low density lipoprotein receptor related protein 1 (LRP1) and activates its downstream signals, including ERK in a ligand specific manner [34]. Here we test the hypothesis that MSCs stimulate tPA expression in astrocytes and activate neuronal LRP1 and ERK, which thereby enhances axonal outgrowth.

## Materials and Methods

All experimental procedures were carried out in accordance with the NIH Guide for the Care and Use of Laboratory Animals and approved by the Institutional Animal Care and Use Committee of Henry Ford Hospital. Animals were maintained on a 12/12 hour Light/Dark cycle with food and water available ad libitum.

### Isolation and culture of primary neurons and astrocytes

Wild type (WT, B57BL/6J) and tPA knockout (KO, with C57BL/6J background) mice (2–3 month-old, purchased from Jackson Laboratories, Bar Harbor, ME) were paired, respectively, for the first two hours of the light period and plug positive mice were separated. The day of plug detection was considered to be embryonic day 0 (E0). Cortical cells were dissected from day 17–18 (E17-18) mouse embryos according to our established procedure with some modifications [35, 36]. Briefly, embryos were removed under deep Ketamine anesthesia, and the cerebral cortex dissected, stripped of meninges, and dissociated by a combination of Ca^2+^ and Mg^2+^ free Hanks balance salt solution (HBSS, Thermo Fisher Scientific Inc. Wayne, MI) containing 0.125% trypsin (Thermo Fisher Scientific Inc.) at 37°C for 20 min, then mechanically triturated for ~20 times. The triturated cells were passed through a 40 μm cell strainer (BD Falcon) and counted. For neuron isolation, cells were cultured in neurobasal growth medium (Thermo Fisher Scientific Inc.) containing 2% B-27 (Thermo Fisher Scientific Inc.), 2 mM GlutaMax (Thermo Fisher Scientific Inc.), and 1% antibiotic-antimycotic (Thermo Fisher Scientific Inc.) (Neurobasal/B27/Glu/AA) in a moist incubator at 37°C/5% CO_2_. For astrocyte isolation, cortical cells were cultured and purified in high glucose Dulbecco's modified eagle medium (DMEM, Thermo Fisher Scientific Inc.) containing 20% FBS (Thermo Fisher Scientific Inc.), 2 mM glutamin and 1% antibiotic-antimycotic (DMEM/Glu/AA/20% FBS) in T-75 tissue culture flasks (Corning St. Louis, MO) in a moist incubator at 37°C/5% CO_2_ as reported [32]. Astrocytes at passage 1 (P1) were stored in liquid nitrogen and P2-3 astrocytes were used for studies. The growth media was changed every other day thereafter.

### Primary MSC culture

Wild type mouse MSCs (P9) were purchased from Cognate Inc. (Fremont, CA), and cultured in Complete Stem Cell Medium (Stem Cell Technology, Vancouver, Canada) and P11-13 cells were used for co-culture experiment.

### Co-culture of axons, MSCs and astrocytes in microfluidic chambers

To separate axons from neuronal soma, a microfluidic chamber (Standard Neuron Device, 450 um microgroove barrier, Cat# SND450, Xona Microfluidics, Temecula, CA) was employed. The small dimension of the microgrooves in the chamber allows axons to sprout from the cell seeded compartment (soma side) into the other compartment of the chamber (axon side), but prevents the passage of cell bodies [37]. Briefly, cleaned, sterilized, and dried chambers were affixed to myelin (10 μg/ml, Sigma St. Louis, MO) and Poly-D-lysine (1 mg/ml, Sigma) coated 6-well plates. The cortical neurons were counted to obtain a density of 3×10^7^ cells/ml, seeded into soma side at a number of 6×10^5^ cells/chamber in DMEM with 5% FBS and incubated in a moist incubator at 37°C/5% CO_2_ for an initial 6 hrs. Then the cells were washed and cultured in Neurobasal/B27/AA/Glu medium. Three days later, astrocytes (4x10^4^/chamber, 2 million/ml), MSCs (4x10^4^/chamber, 2 million/ml) or both astrocytes (4x10^4^/chamber, 2 million/ml) and MSCs (800/chamber, 4x10^4^/ml, ratio of MSC to astrocyte = 1/50) were co-seeded in axon side of microfluidic in Neurobasal/B27/Glu/AA containing 2% FBS (Neurobasal/B27/Glu/AA/2% FBS). U0126 (50 μM, InvivoGen), an ERK inhibitor or tPA (10–100 nM, Activase, Genentech, CA) were included in the media in some chambers. The growth media was changed every other day thereafter.

### Oxygen deprivation of neurons

Three days after seeded in microfluidic chamber, neurons in Neurobal/B27/Glu/AA were cultured in an enclosed anaerobic chamber (Model 1025, Forma Scientific, Marietta OH) at 37°C for 2 hrs. Then the neurons were exposed to normal culture conditions for the following experiments until sample collection [32, 38]. This chamber maintains strict anaerobiosis to less than 10 μg/mL O_2_ (according to the specifications provided by the manufacturer). The oxygen level within the chamber was routinely measured with a BD Disposable Anaerobic Indicator (Becton, Dickinson and Company, Sparks, MD), which confirmed that the oxygen level remained below 0.2% [32, 38].

### Axon Immunostaining and quantification

Five days after primary cortical cells were seeded, neurons in the microfluidic chamber were fixed by 4% paraformaldehyde, incubated with anti-Tuj-1 antibody (1:500, Covance Princeton, NJ) overnight and followed with Cy3 labeled secondary antibody. Nuclei were counter-stained by DAPI (1:10000, Thermo Fisher Scientific Inc). Axons were recognized by Tuj-1 positive fibers and quantified with ImageJ software 1.34 to show total axon length (μM) in a field. To rule out possible dendrite contamination in microgrooves, we selected cross-line between the ends of microgrooves and axonal compartment as the reference line and do binning through the entire axonal compartment. At least fifty randomly selected axon fields in more than 8 microfluidic chambers from 3 different experiments per group were quantified by experimenters blinded to each culture condition.

### Co-culture of astrocytes and MSCs

WT murine astrocytes (P2-3) and MSCs (P11-13) were individually cultured in 6-cm dishes with DMEM/Glu/AA/20% FBS at 37°C/5% CO_2_. When astrocytes reached 50–60% confluence, MSCs were harvested and seeded into astrocyte dishes (0.5 million/dish) and co-cultured in DMEM/Glu/AA/20% FBS at 37°C/5% CO_2_. When the astrocytes and/or MSCs reached 80–90% confluence (3 groups, astrocyte, MSC and astrocyte+MSC), they were harvested for Western blot against tPA.

### Co-culture of neurons, MSCs and astrocytes in plates and cell culture inserts

Six well plates (Corning, Corning, NY) were coated with poly-D-lysine (25 μg/ml) at 37°C overnight. WT-cortical cells (1x10^6^/well in 2ml) were seeded in the 6-well plates and cultured in Neurobasal/B27/Glu/AA culture medium for 3 days. Then growth medium were changed to Neurobasal/B27/Glu/AA/2% FBS. MSC (0.2x10^6^/well in 1 ml), WT-astrocytes (0.2x10^6^/well in 1ml) or both (MSC 4000/well & WT-astrocytes 0.2x10^6^/well respectively in 1ml, ratio of MSC to astrocyte = 1/50) were seeded into cell culture inserts (Corning) matching the 6-well plates. Inserts and cortical cells in wells were co-cultured in Neurobasal/B27/Glu/AA/2% FBS. Receptor related protein, RAP (50 nM), a ligand antagonist of low density lipoprotein receptor related protein-1 (LRP1) or U0126 (50 μM) were added to some wells to test the involvement of LRP1 or ERK. Neurons were harvested for Western blot against tPA and p-ERK/ERK after 3 days.

### Western blot assay

Cells were rinsed with PBS, and then lysed in the RIPA lysis buffer (Sigma) containing proteinase inhibitor cocktail-1 (Calbiochem, Billerica, MA) and phosphatase inhibitors cocktail-2 (Sigma, P5726-1ml). Protein concentrations were determined using the Bicinchoninic Acid (BCA) protocol (Pierce, Rockford, IL). 30 μg total protein was loaded on 10% Bis-Tris Gels (Invitrogen, San Diego, CA) for Western blot assay following the standard Western blotting protocol (Molecular Clone, Edition II). Primary antibodies were employed, including tPA (1:1000, Abcam, Cambridge, MA), ERK-1 (1:1000, Santa Cruz, Dallas, Texas), Phospho-ERK (1:1000, Santa Cruz, Dallas, Texas) and β-actin (1:5000, Abcam). Respective horseradish peroxidase (HRP) labeled secondary antibodies were applied and enhanced chemiluminescence (ECL) detection was used to detect target bands according to the manufacturer's instructions (Pierce, Rockford, IL). The integrated density mean grey value of the bands was analyzed under ImageJ software. tPA/b-actin and p-ERK/ERK-1 relative expression ratio was calculated.

### Statistics

Data are expressed as Means±SE. The differences between mean values were evaluated with the two tailed Student's t-test (for 2 groups) and the analysis of variance (ANOVA, for >2 groups). All calculations and statistical tests employed Microsoft Excel 2013 (Microsoft, Redmond, WA) or SPSS 11.5 (SPSS, Chicago, IL). P<0.05 was considered significant for all analyses.

## Results

### MSCs promote axonal outgrowth alone and synergistically with astrocytes under normoxia conditions

**Fig 1:**
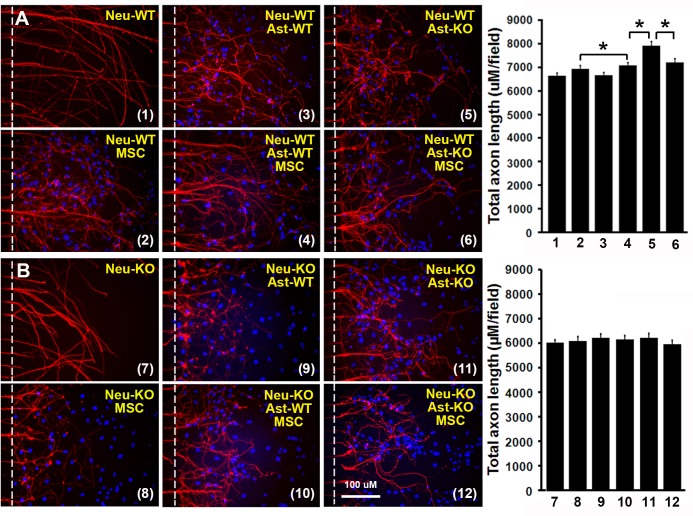
MSCs promote axonal outgrowth via tPA alone and synergistically with astrocytes at normoxia. Primary neurons were cultured in microfluidics chambers for 3 days at normoxia. Astrocytes and/or MSCs were seeded in the axon side of the chambers and cultured for another 2 days at normoxia. Total axon length (μM/field) was quantified and compared. **(A)**: Representative immunofluorescent images of WT axons from co-culture of WT axons with MSCs and/or astrocytes (WT or tPA-KO) and quantitative data. **(B):** Representative immunofluorescent images of tPA-KO axons from co-culture of tPA-KO axons with MSCs and/or astrocyte (WT or tPA-KO) and quantitative data. N = 10-12/group. Scale bar = 100 μm. Dash lines show the borders to quantify the total axon length in the axonal compartments. *p<0.05. Neu = neuron, Ast = astrocyte, WT = wild type, KO = tPA knockout. MSC = mesenchymal stromal cell. 1: Neu-WT; 2: Neu-WT+ast-WT; 3: Neu-WT+ast-KO; 4: Neu-WT+MSC; 5: Neu-WT+MSC+ast-WT; 6: Neu-WT+MSC+ast-KO; 7: Neu-KO; 8: Neu-KO+ast-WT; 9: Neu-KO+ast-KO; 10: Neu-KO+MC; 11: Neu-KO+MSC+ast-KO and 12: Neu-KO+MSC+ast-KO.

To test the effect of MSCs on axonal outgrowth, we co-cultured MSCs and/or astrocytes (tPA-KO and WT) with WT-axons in microfluidic chambers under normoxia cell culture conditions for 3 days. Axonal outgrowth under different conditions was evaluated with the immunostaining fluorescent positive fiber length measured using ImageJ, shown as total axonal length in a randomly-selected field. As shown in Fig 1A, neither WT nor KO astrocytes directly enhance total WT-axonal length (6937±133 μM/field, 6657±119 μM/field and 6643±112 μM/field, respectively, n = 10-11/group, P>0.05). However, MSCs promoted total WT-axonal length from 6643±112 μM/field to 7070±133 μM/field directly (n = 10–11, P<0.05) and synergistically with WT-astrocytes from 7070±133 μM/field to 7910±196 μM/field (n = 8-10/group, P<0.05), but there was no additive effect with KO-astrocytes (7070±133 μM/field vs 7210±147 μM/field, n = 8-10/group, P>0.05), suggesting that astrocytic tPA partially mediates the synergistic promoting effect of MSC. We also performed co-culture of MSCs and/or astrocytes (KO and WT) with tPA-KO axons in parallel experiments (Fig 1B). No difference was found in total axonal length between all the 6 groups (neuron-KO:6027±105 μM/field, neuron-KO+astrocyte-WT:6090±175 μM/field, neuron-KO+astrocyte-KO:6216±154 μM/field, neuron-KO+MSC:6153±147 μM/field, neuron-KO+MSC+astrocyte-WT:6223±161 μM/field and neuron-KO+MSC+astrocyte-KO:5964±147 μM/field, respectively, n = 10-12/group, P>0.05), suggesting that neuronal tPA is also important for MSC promoted axonal outgrowth.

### MSCs promote axonal outgrowth alone and synergistically with astrocytes under oxygen deprivation conditions

**Fig 2:**
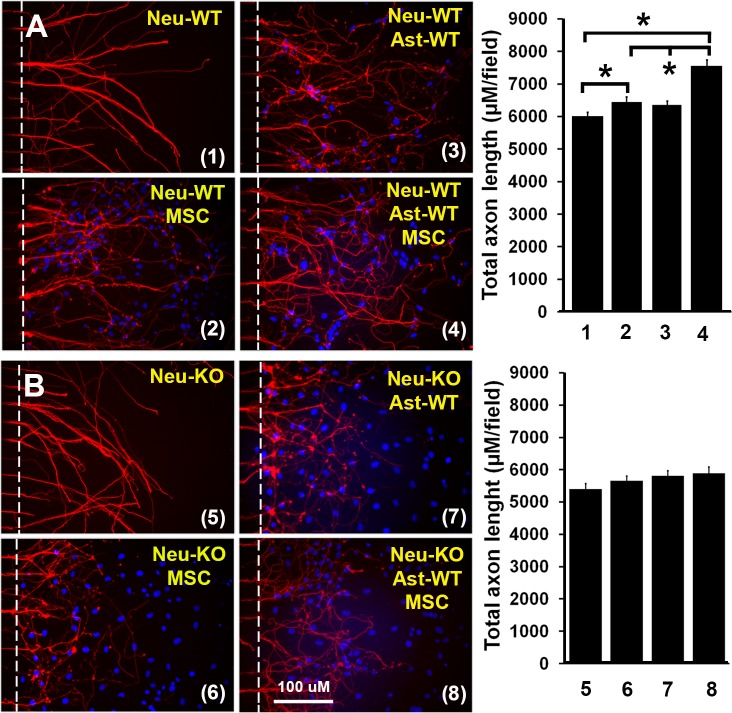
MSCs promoted axonal outgrowth via tPA alone and synergistically with astrocytes under conditions of oxygen deprivation. Primary neurons were cultured in microfluidic chambers for 3 days at normoxia. Then, the microfluidic chambers were moved into an anaerobic chamber for 2 hrs. Astrocytes and/or MSCs were seeded in axon side of the chambers and cultured for another 2 days at normoxia. Total axon length (μM/field) was quantified and compared. **(A):** Representative immunofluorescent images of WT axons from co-culture of WT axons with MSCs and/or WT astrocytes and quantitative data at oxygen deprivation. **(B)**: Representative immunofluorescent images of tPA-KO axons from co-culture of tPA-KO axons with MSCs and/or tPA-KO astrocytes and quantitative data at oxygen deprivation. N = 10-12/group. Scale bar = 100 μm. *p<0.05. Neu = neuron, Ast = astrocyte, MSC = mesenchymal stromal cell, WT = wild type, KO = tPA knockout. 1: Neu-WT, 2: Neu-WT+MSC, 3: Neur-WT, 4: Neu-WT+Ast-WT+MSC, 5: Neu-KO, 6: Neu-KO+MSC, 7: Neu-KO+At-WT and 8: Neu-KO+Ast-WT+MSC.

To test whether MSCs enhance axonal outgrowth under conditions that may reflect ischemic stroke, the same experiments as above performed under normoxia conditions, were now performed under conditions where axons in the microfluidic chambers were subjected in oxygen deprivation (OD). Data were similar to those obtained employing normoxia axonal culture (Fig 2). WT astrocytes did not directly enhance total hypoxic WT axonal length (6363±112 μM/field vs 6013±119 μM/field, n = 10-12/group, P>0.05). MSCs alone increased total WT axonal length from 6013±119 μM/field to 6454±147 μM/field (n = 10-12/group, P<0.05); MSC plus WT astrocytes synergistically increased the total axonal length from 6454±147 μM/field to 7560±182 μM/field (n = 10-12/group, P<0.05), indicating that the beneficial effect of MSC’s is retained for axons under hypoxic conditions. Similar to normoxia data, MSCs exerted no effect on total axonal length of KO axons under hypoxic conditions (neuron-KO:5397±168 μM/field, neuron-KO+MSC:5663±140 μM/field, neuron-KO+astrocyte-KO:5817±154 μM/field and neuron-KO+MSC+astrocyte-KO:5894±189 μM/field, respectively, n = 10-12/group, P>0.05). We also co-cultured KO-astrocytes with WT-neurons/MSCs and WT-astrocytes with KO-neurons/MSC, and obtained similar results to normoxia axonal culture (data not shown).

### Both endogenous and exogenous tPA promote axonal outgrowth

**Fig 3:**
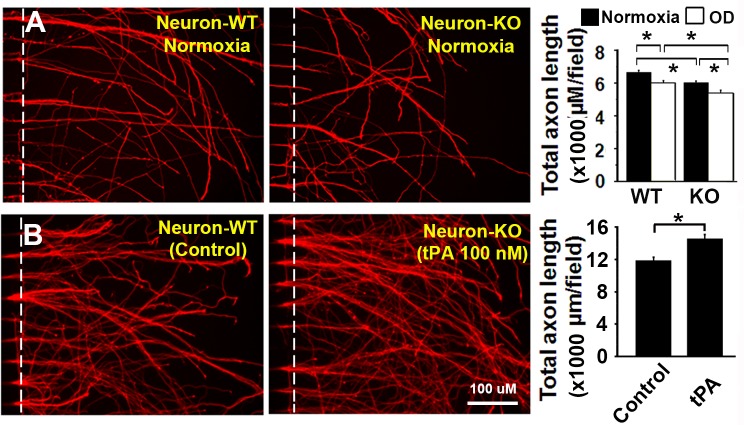
Both neuronal and exogenous tPA promoted axonal outgrowth. **(A)**: Neuronal tPA promoted axonal outgrowth. Primary WT and tPA-KO neurons were cultured at both normoxia and oxygen deprivation. Total axon length (μM/field) was quantified and compared. N = 10-11/group. **(B)**: Exogenous tPA promoted axonal outgrowth. Primary WT neurons were cultured in medium containing tPA (100 nM) and total axon length (μM/field) was quantified and compared. N = 5-6/group. Scale bar = 100 μM. *p<0.05.

To investigate the role of neuronal endogenous tPA on axonal outgrowth, both WT and tPA-KO neurons were, respectively, cultured under normoxia and oxygen deprivation conditions in microfluidic chambers. As shown in Fig 3A, total axonal length from WT neurons was significantly longer than the length from KO neurons at both normoxia (6650±112 μM/field vs 6020±105 μM/field, n = 10-11/group, P<0.05) and oxygen deprivation (6020±119 μM/field vs 5390±168 μM/field, n = 10/group, P<0.05) conditions, suggesting that neuronal endogenous tPA contributes to axonal outgrowth even for hypoxic neurons. To test the effect of exogenous tPA, neurons were seeded and tPA (10, 30 and 100 nM) was added to the medium of Neurobasal/B27/AA/Glu medium in microfluidic chambers throughout the 5-day culture period. tPA increased total axonal length dose-dependently (data not shown). As shown in Fig 3B, tPA (100 nM) increased total axonal length significantly from 11900±385 μM/field to 14560±476 μM/field (n = 5-6/group, P<0.05).

### MSCs increase tPA expression in both astrocytes and neurons

**Fig 4:**
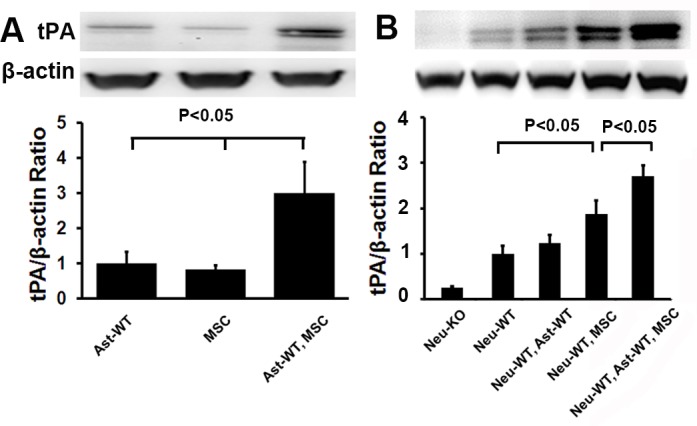
MSCs increased tPA in both astrocytes and neurons. **(A):** MSCs increased astrocytic tPA. WT astrocytes and MSCs were cultured individually and together for cellular tPA by Western blot. Similar levels of tPA were detected from both astrocytes and MSCs. Co-culture of astrocytes and MSCs increased tPA expression significantly (N = 6/group, P<0.05). **(B)**: MSCs increased neuronal tPA directly and synergistically with astrocytes. WT neurons in plates were co-cultured with MSCs and/or astrocytes seeded in cell culture inserts in culture medium for neuronal tPA by Western blot. N = 6/group. Representative Western blot for tPA and quantification data were shown. WT = wild type, KO = tPA KO, Ast = astrocyte, MSC = mesenchymal stromal cell, Neu = neuron.

To test whether tPA expression in astrocytes and neurons is altered by co-culture with MSCs, Western blot was carried out. MSCs and WT-astrocytes were co-cultured in dishes for 3 days and then Western blot was performed to detect intracellular tPA. As shown in Fig 4A, co-culture with MSCs significantly increased astrocytic tPA expression approximately 3 fold (n = 6/group, P<0.05), in agreement with our previous publication that MSCs increase astrocytic tPA [32].

Next, WT-neurons were plated for 3 days and then co-cultured with MSCs and/or WT astrocytes seeded in cell culture inserts. Three days later, neurons were harvested and tPA was quantified by Western blot. As seen in Fig 4B, astrocytes alone did not increase WT neuronal tPA (1.2 vs 1.0, n = 6/group, P>0.05). MSCs increased neuronal tPA nearly 2 fold (P<0.05) and MSCs plus astrocytes increased tPA nearly 3 fold (P<0.05), indicating that MSCs increase neuronal tPA expression directly and synergistically with astrocytes.

### MSCs increase axonal outgrowth via LRP1 and ERK pathway

**Fig 5:**
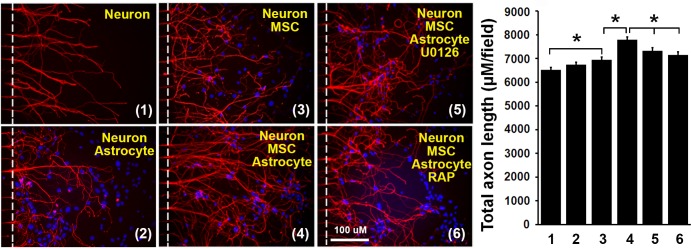
MSCs promoted axonal outgrowth via LRP1 and ERK pathway. Axons were cultured individually and co-cultured with WT-astrocytes and/or MSC in microfluidic chambers in culture medium or media containing RAP (50 nM) or U0126 (50 μM). Total axon length (μM/field) was quantified and compared. Representative images and quantification data are shown. N = 10-12/group. Scale bar = 100 μm. *p<0.05. RAP = Receptor related protein. MSC = mesenchymal stromal cell. 1: Neuron, 2: Neuron+Astrocyte, 3: Neuron+MSC, 4: Neuron+MSC+Astrocyte, 5: Neuron+MSC+Astrocyte+U0126, 6: Neuron+MSC+Astrocyte.

To investigate signaling pathways underlying the MSC promotion of axonal outgrowth, RAP (an antagonist of LRP1 which blocks tPA binding to LRP1) or U0126 (an ERK inhibitor) were added to the axon side of the microfluidic chambers, in which WT axons were co-cultured with WT astrocytes and/or MSCs. As illustrated in Fig 5, astrocytes alone did not increase total WT axonal length (6734±112 μM/field vs 6517±98 μM/field, n = 10/group, P>0.05). MSCs alone increased total axonal length from 6517±98 μM/field to 6944±119 μM/field (n = 10-11/group, P<0.05). Co-culture of astrocytes, MSCs and axons increased total axonal length further from 6944±119 μM/field to 7777±133 μM/field (n = 10, P<0.05), which was inhibited by RAP (50 nM) from 7777±133 μM/field to 7147±119 μM/field (n = 10, P<0.05) and by U0126 (50 μM) from 7777±133 μM/field to 7315±133 μM/field (n = 9, P<0.05). These data demonstrate that the MSC promotion of axonal outgrowth via astrocytic tPA is LRP1 and ERK dependent.

### MSCs activate neuronal ERK via tPA binding to LRP1

**Fig 6:**
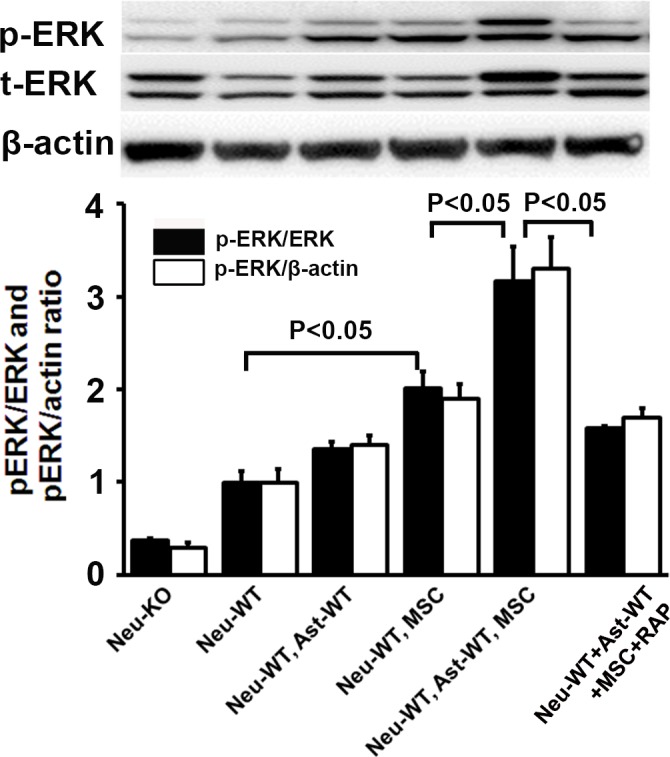
MSCs activated neuronal ERK via tPA binding to LRP1. WT neurons in plates were co-cultured with MSCs and/or astrocytes seeded in cell culture inserts in culture medium or media containing RAP (50 nM). Representative Western blot for p-ERK, ERK and quantification data are shown. N = 6/group. RAP = Receptor associated protein. Neu = neuron, WT = wild type, KO = tPA knockout, Ast = Astrocyte, MSC = mesenchymal stromal cell.

To investigate neuronal ERK activation following MSC treatment, WT neurons were cultured in plates for 3 days. Then, MSCs and/or WT astrocytes seeded in cell culture inserts were co-cultured with the neurons in Neurobasal/B27/AA/Glu/2% FBS media and this media containing RAP (50 nM), respectively. Three days later, neurons were harvested, and p-ERK and ERK were quantified by Western blot. As shown in Fig 6, MSCs activated neuronal ERK 1 fold higher (n = 6/group, P<0.05), and MSCs plus WT astrocytes elevated p-ERK more than 2 fold (P<0.05). However, the combination of MSCs plus WT astrocytes mediated increase was inhibited by RAP (P<0.05), suggesting that MSC induced ERK activation is dependent on upstream tPA binding to LRP1 receptor.

## Discussion

Axons play a central role in both the injury and repair phases after stroke [39]. The microfluidic chamber provides an excellent system for separating soma and dendrites from axons [35–37]. Axons in the chamber can be individually studied without interference from soma or dendrites. We are the first to co-culture MSCs, astrocytes and axons in a microfluidic chamber and investigate the effects of MSCs and astrocytes exclusively on axonal outgrowth.

Our data demonstrate that MSCs promote WT axonal outgrowth alone and synergistically with WT astrocytes, but not with tPA-KO astrocytes (Fig 1A); however, MSCs showed no effect on tPA-KO axonal outgrowth even with astrocyte (WT and KO) co-culture (Fig 1B), indicating that MSCs increase axonal outgrowth by both neuron and astrocyte tPA, and the expression of tPA in neurons is critical to observe the synergistic effect of MSCs and astrocytes on axonal outgrowth. When the same experiments were performed under oxygen deprivation conditions (an in vitro condition mimic to brain ischemia) [32, 38, 40], the MSC promoting effect on axonal outgrowth remained intact (Fig 2), suggesting that MSCs may provide benefit for patients of ischemic stroke via tPA. In addition, we compared axonal outgrowth from WT and tPA-KO neurons, and found that WT axons exhibited increased total axonal length compared to tPA-KO axons. This further confirmed that neuronal tPA contributes to axonal outgrowth (Fig 3A). Then we cultured neurons with tPA in the medium and observed that exogenous non-neuronal tPA also promoted axonal outgrowth, consistent with the data of Fig 1, that astrocytic tPA mediates MSC-induced axonal outgrowth. Co-culture of MSCs with astrocytes significantly increased tPA expression in astrocytes compared with astrocyte cell culture alone (Fig 4A), which is in agreement with prior studies that MSCs increase tPA expression and secretion from astrocytes in vitro [32].

MSCs modulate endogenous tPA level and activity in the ischemic boundary zone in mice subjected to middle cerebral artery occlusion and tPA plays a pivotal role in neurite outgrowth [18, 32]. The sonic hedgehog (Shh) pathway mediates the overexpression of tPA in neurons and astrocytes of ischemic boundary zone after MSC transplantation [41]. In the central nervous system, tPA is the major plasminogen activator and tPA activity is mainly inhibited by plasminogen activator inhibitor -1 (PAI-1) [42, 43]. tPA promotes brain plasticity via its proteolytic and non-proteolytic pathways [44–46]. As the proteolytic function, tPA cleaves the precursor forms of neurotrophins, for example, pro-BDNF and pro-NGF, to the active forms of BDNF and NGF, respectively [47, 48]. These active neurotrophins promote neurite remodeling [49–53]. Thus, it is reasonable to expect that the individually promoting effect of MSCs on axonal outgrowth may be mediated through tPA proteolytic function.

tPA also acts by the non-proteolytic pathways. Recently reported, tPA combines with the LRP1 receptor in PC12 and N2a neuron-like cells to initiate downstream signaling of ERK in a biphasic manner [34]. However, combination of myelin-associated glycoprotein (MAG) with LRP1 did not activate the ERK pathway, suggesting a mechanism of tPA-ligand-specific co-receptor recruitment of LRP1 [34, 54].

LRP1 is a type-1 transmembrane receptor that binds to more than forty distinct ligands [55] and is widely localized to axons and neuronal growth cones, in intracellular vesicles and at the cell surface of neurons in nervous system [56–58]. LRP1 regulates cell-signaling in conjunction with diverse co-receptors, including N-methyl-D-aspartate receptor (NMDA receptor), tyrosine kinase receptors (Trk receptors), urokinase-type plasminogen activator receptor (uPAR), tumor necrosis factor receptor 1 (TNFR1) and platelet derived growth factor receptor (PDGF receptor) [34, 59–63]. Hence, the activity of LRP1 in cell-signaling may be ligand-specific. In neurons and neuron-like cell lines, binding of tPA to LRP1 activates ERK and AKT to promote neurite outgrowth [63–65]. RAP is a molecular chaperone for LRP1 and has been extensively used to inhibit the functional activity of LRP1 [66, 67]. As a potent LRP1 antagonist, exogenously added RAP binds to LRP1 on the cell surface, preventing ligands, e.g. tPA, from binding [34, 66–68].

In the present study, we concentrated on non-proteolytic tPA pathways. To investigate the synergistic promoting effects of tPA on axonal outgrowth, the ERK inhibitor, U0126 and the LRP1 inhibitor, RAP, were included in the microfluidics chambers. As shown in Fig 5, MSCs enhanced axonal outgrowth alone. Co-culture of MSCs, astrocytes and axons synergistically further enhanced axonal outgrowth, which was inhibited by RAP and U0126, indicating that the synergistic effect was mediated by LRP1 receptor and was ERK-dependent. We have reported that MSCs increase tPA expression and secretion from astrocytes in vitro [32, 33]. We therefore speculate, that MSCs enhance secretion of astrocytic tPA which is transported to axonal membranes, possibly via exosomes, and which combines with LRP1 resulting in neuronal ERK activation and axonal outgrowth [34, 64].

Technically, it is difficult to test ERK activation in axons with microfluidic model, since axons were co-cultured with astrocytes and/or MSCs in axonal compartments. Instead, neuronal ERK activation was studied by Western blot. The Western data were not specifically axonal, but are consistent with our data of axonal growth and support our hypothesis. In Fig 6, MSCs activated neuronal ERK alone and synergistically with astrocytes. The activation was inhibited by RAP, indicating that the ERK activation is dependent on tPA binding to LRP1. Significant differences in ERK activation were detected between WT neurons and tPA-KO neurons before co-culture with other cells (Fig 6). Possibly, neuronal tPA from WT-neurons comes out of cytoplasm (e.g. exocytosis via exosomes) and then combines LRP1, resulting in activation of neuronal ERK [34, 64, 69, 70]. Further investigation is warranted.

## Conclusions

In summary, our data indicate that MSCs stimulate axonal outgrowth alone by neuronal tPA and synergistically with astrocytic tPA. Neuronal tPA is critical to observe the synergistic effect of MSC and astrocytes on axonal outgrowth. The increased tPA expression from astrocytes (and neurons) may bind to the neuronal membrane LRP1 receptor, which activates downstream ERK and thereby promotes axonal outgrowth.

## References

[1] Alberts MJ, Naidech AM. tPA and warfarin: time to move forward. Neurology. 2013;80(6):514–5. 10.1212/WNL.0b013e31827b1b7c 23223535

[2] Fang MC, Cutler DM, Rosen AB. Trends in thrombolytic use for ischemic stroke in the United States. J Hosp Med. 2010;5(7):406–9. PubMed Central PMCID: PMC3024589. 10.1002/jhm.689 20578049 PMC3024589

[3] Ibrahim F, Akhtar N, Salam A, Kamran S, Deleu D, D'Souza A, et al Stroke Thrombolysis Protocol Shortens "Door-to-Needle Time" and Improves Outcomes-Experience at a Tertiary Care Center in Qatar. J Stroke Cerebrovasc Dis. 2016. 10.1016/j.jstrokecerebrovasdis.2016.03.047 27256170

[4] Eckert MA, Vu Q, Xie K, Yu J, Liao W, Cramer SC, et al Evidence for high translational potential of mesenchymal stromal cell therapy to improve recovery from ischemic stroke. J Cereb Blood Flow Metab. 2013;33(9):1322–34. PubMed Central PMCID: PMCPMC3764389. 10.1038/jcbfm.2013.91 23756689 PMC3764389

[5] Gutierrez-Fernandez M, Otero-Ortega L, Ramos-Cejudo J, Rodriguez-Frutos B, Fuentes B, Diez-Tejedor E. Adipose tissue-derived mesenchymal stem cells as a strategy to improve recovery after stroke. Expert Opin Biol Ther. 2015;15(6):873–81. 10.1517/14712598.2015.1040386 25959243

[6] Zhang L, Zhang RL, Wang Y, Zhang C, Zhang ZG, Meng H, et al Functional recovery in aged and young rats after embolic stroke: treatment with a phosphodiesterase type 5 inhibitor. Stroke. 2005;36(4):847–52. 10.1161/01.STR.0000158923.19956.73 15746452

[7] Chopp M, Li Y, Zhang ZG. Mechanisms underlying improved recovery of neurological function after stroke in the rodent after treatment with neurorestorative cell-based therapies. Stroke. 2009;40(3 Suppl):S143–5. PubMed Central PMCID: PMCPMC2854491. 10.1161/STROKEAHA.108.533141 19064763 PMC2854491

[8] Honmou O, Onodera R, Sasaki M, Waxman SG, Kocsis JD. Mesenchymal stem cells: therapeutic outlook for stroke. Trends Mol Med. 2012;18(5):292–7. 10.1016/j.molmed.2012.02.003 22459358

[9] Bang OY, Lee JS, Lee PH, Lee G. Autologous mesenchymal stem cell transplantation in stroke patients. Ann Neurol. 2005;57(6):874–82. 10.1002/ana.20501 15929052

[10] Bhasin A, Srivastava MV, Kumaran SS, Mohanty S, Bhatia R, Bose S, et al Autologous mesenchymal stem cells in chronic stroke. Cerebrovasc Dis Extra. 2011;1(1):93–104. PubMed Central PMCID: PMCPMC3343764. 10.1159/000333381 22566987 PMC3343764

[11] Hermann DM, Chopp M. Promoting brain remodelling and plasticity for stroke recovery: therapeutic promise and potential pitfalls of clinical translation. Lancet Neurol. 2012;11(4):369–80. PubMed Central PMCID: PMCPMC3964179. 10.1016/S1474-4422(12)70039-X 22441198 PMC3964179

[12] Li Y, Chopp M. Marrow stromal cell transplantation in stroke and traumatic brain injury. Neurosci Lett. 2009;456(3):120–3. PubMed Central PMCID: PMCPMC3359793. 10.1016/j.neulet.2008.03.096 19429146 PMC3359793

[13] Li Y, Liu Z, Xin H, Chopp M. The role of astrocytes in mediating exogenous cell-based restorative therapy for stroke. Glia. 2014;62(1):1–16. PubMed Central PMCID: PMCPMC3947888. 10.1002/glia.22585 24272702 PMC3947888

[14] Li Y, Chen J, Zhang CL, Wang L, Lu D, Katakowski M, et al Gliosis and brain remodeling after treatment of stroke in rats with marrow stromal cells. Glia. 2005;49(3):407–17. 10.1002/glia.20126 15540231

[15] Li Y, McIntosh K, Chen J, Zhang C, Gao Q, Borneman J, et al Allogeneic bone marrow stromal cells promote glial-axonal remodeling without immunologic sensitization after stroke in rats. Exp Neurol. 2006;198(2):313–25. 10.1016/j.expneurol.2005.11.029 16455080

[16] Liu Z, Li Y, Zhang ZG, Cui X, Cui Y, Lu M, et al Bone marrow stromal cells enhance inter- and intracortical axonal connections after ischemic stroke in adult rats. J Cereb Blood Flow Metab. 2010;30(7):1288–95. PubMed Central PMCID: PMCPMC2896436. 10.1038/jcbfm.2010.8 20125183 PMC2896436

[17] Chen J, Li Y, Wang L, Zhang Z, Lu D, Lu M, et al Therapeutic benefit of intravenous administration of bone marrow stromal cells after cerebral ischemia in rats. Stroke. 2001;32(4):1005–11. 11283404 10.1161/01.str.32.4.1005

[18] Shen LH, Xin H, Li Y, Zhang RL, Cui Y, Zhang L, et al Endogenous tissue plasminogen activator mediates bone marrow stromal cell-induced neurite remodeling after stroke in mice. Stroke. 2011;42(2):459–64. PubMed Central PMCID: PMC3093714. 10.1161/STROKEAHA.110.593863 21212396 PMC3093714

[19] Chen J, Li Y, Katakowski M, Chen X, Wang L, Lu D, et al Intravenous bone marrow stromal cell therapy reduces apoptosis and promotes endogenous cell proliferation after stroke in female rat. J Neurosci Res. 2003;73(6):778–86. 10.1002/jnr.10691 12949903

[20] Barzilay R, Ganz J, Sadan O, Ben-Zur T, Bren Z, Hinden N, et al Mesenchymal stem cells protect from sub-chronic phencyclidine insult in vivo and counteract changes in astrocyte gene expression in vitro. Eur Neuropsychopharmacol. 2013;23(9):1115–23. 10.1016/j.euroneuro.2012.10.002 23116946

[21] Gao Q, Li Y, Chopp M. Bone marrow stromal cells increase astrocyte survival via upregulation of phosphoinositide 3-kinase/threonine protein kinase and mitogen-activated protein kinase kinase/extracellular signal-regulated kinase pathways and stimulate astrocyte trophic factor gene expression after anaerobic insult. Neuroscience. 2005;136(1):123–34. 10.1016/j.neuroscience.2005.06.091 16198497

[22] Filous AR, Silver J. "Targeting astrocytes in CNS injury and disease: A translational research approach". Prog Neurobiol. 2016. 10.1016/j.pneurobio.2016.03.009 PMC5035184 27026202

[23] Powell EM, Meiners S, DiProspero NA, Geller HM. Mechanisms of astrocyte-directed neurite guidance. Cell Tissue Res. 1997;290(2):385–93. 9321702 10.1007/s004410050945

[24] Cramer SC, Nudo RJ. Brain repair after stroke Cambridge: New York: Cambridge University Press; 2010 x, 292 p. p.

[25] Chen J, Zhang ZG, Li Y, Wang L, Xu YX, Gautam SC, et al Intravenous administration of human bone marrow stromal cells induces angiogenesis in the ischemic boundary zone after stroke in rats. Circ Res. 2003;92(6):692–9. 10.1161/01.RES.0000063425.51108.8D 12609969

[26] Gutierrez-Fernandez M, Rodriguez-Frutos B, Ramos-Cejudo J, Teresa Vallejo-Cremades M, Fuentes B, Cerdan S, et al Effects of intravenous administration of allogenic bone marrow- and adipose tissue-derived mesenchymal stem cells on functional recovery and brain repair markers in experimental ischemic stroke. Stem Cell Res Ther. 2013;4(1):11 PubMed Central PMCID: PMCPMC3706777. 10.1186/scrt159 23356495 PMC3706777

[27] Wakabayashi K, Nagai A, Sheikh AM, Shiota Y, Narantuya D, Watanabe T, et al Transplantation of human mesenchymal stem cells promotes functional improvement and increased expression of neurotrophic factors in a rat focal cerebral ischemia model. J Neurosci Res. 2010;88(5):1017–25. 10.1002/jnr.22279 19885863

[28] Alder J, Kramer BC, Hoskin C, Thakker-Varia S. Brain-derived neurotrophic factor produced by human umbilical tissue-derived cells is required for its effect on hippocampal dendritic differentiation. Dev Neurobiol. 2012;72(6):755–65. 10.1002/dneu.20980 21954108

[29] Kurozumi K, Nakamura K, Tamiya T, Kawano Y, Ishii K, Kobune M, et al Mesenchymal stem cells that produce neurotrophic factors reduce ischemic damage in the rat middle cerebral artery occlusion model. Mol Ther. 2005;11(1):96–104. 10.1016/j.ymthe.2004.09.020 15585410

[30] Chen X, Li Y, Wang L, Katakowski M, Zhang L, Chen J, et al Ischemic rat brain extracts induce human marrow stromal cell growth factor production. Neuropathology. 2002;22(4):275–9. 12564767 10.1046/j.1440-1789.2002.00450.x

[31] Qu R, Li Y, Gao Q, Shen L, Zhang J, Liu Z, et al Neurotrophic and growth factor gene expression profiling of mouse bone marrow stromal cells induced by ischemic brain extracts. Neuropathology. 2007;27(4):355–63. PubMed Central PMCID: PMCPMC2593420. 10.1111/j.1440-1789.2007.00792.x 17899689 PMC2593420

[32] Xin H, Li Y, Shen LH, Liu X, Wang X, Zhang J, et al Increasing tPA activity in astrocytes induced by multipotent mesenchymal stromal cells facilitate neurite outgrowth after stroke in the mouse. PLoS One. 2010;5(2):e9027 PubMed Central PMCID: PMC2815778. 10.1371/journal.pone.0009027 20140248 PMC2815778

[33] Xin H, Li Y, Shen LH, Liu X, Hozeska-Solgot A, Zhang RL, et al Multipotent mesenchymal stromal cells increase tPA expression and concomitantly decrease PAI-1 expression in astrocytes through the sonic hedgehog signaling pathway after stroke (in vitro study). J Cereb Blood Flow Metab. 2011;31(11):2181–8. PubMed Central PMCID: PMCPMC3210339. 10.1038/jcbfm.2011.116 21829213 PMC3210339

[34] Mantuano E, Lam MS, Gonias SL. LRP1 assembles unique co-receptor systems to initiate cell signaling in response to tissue-type plasminogen activator and myelin-associated glycoprotein. J Biol Chem. 2013;288(47):34009–18. PubMed Central PMCID: PMC3837140. 10.1074/jbc.M113.509133 24129569 PMC3837140

[35] Zhang Y, Chopp M, Liu XS, Katakowski M, Wang X, Tian X, et al Exosomes Derived from Mesenchymal Stromal Cells Promote Axonal Growth of Cortical Neurons. Mol Neurobiol. 2016. 10.1007/s12035-016-9851-0 PMC5028236 26993303

[36] Zhang Y, Ueno Y, Liu XS, Buller B, Wang X, Chopp M, et al The MicroRNA-17-92 cluster enhances axonal outgrowth in embryonic cortical neurons. J Neurosci. 2013;33(16):6885–94. PubMed Central PMCID: PMCPMC3657758. 10.1523/JNEUROSCI.5180-12.2013 23595747 PMC3657758

[37] Taylor AM, Jeon NL. Microfluidic and compartmentalized platforms for neurobiological research. Crit Rev Biomed Eng. 2011;39(3):185–200. 21967302 10.1615/critrevbiomedeng.v39.i3.20

[38] Xin H, Chopp M, Shen LH, Zhang RL, Zhang L, Zhang ZG, et al Multipotent mesenchymal stromal cells decrease transforming growth factor beta1 expression in microglia/macrophages and down-regulate plasminogen activator inhibitor 1 expression in astrocytes after stroke. Neurosci Lett. 2013;542:81–6. PubMed Central PMCID: PMCPMC3678739. 10.1016/j.neulet.2013.02.046 23499476 PMC3678739

[39] Hinman JD. The back and forth of axonal injury and repair after stroke. Curr Opin Neurol. 2014;27(6):615–23. PubMed Central PMCID: PMCPMC4459741. 10.1097/WCO.0000000000000149 25364952 PMC4459741

[40] Tabakman R, Jiang H, Shahar I, Arien-Zakay H, Levine RA, Lazarovici P. Neuroprotection by NGF in the PC12 in vitro OGD model: involvement of mitogen-activated protein kinases and gene expression. Ann N Y Acad Sci. 2005;1053:84–96. 10.1196/annals.1344.008 16179511

[41] Ding X, Li Y, Liu Z, Zhang J, Cui Y, Chen X, et al The sonic hedgehog pathway mediates brain plasticity and subsequent functional recovery after bone marrow stromal cell treatment of stroke in mice. J Cereb Blood Flow Metab. 2013;33(7):1015–24. PubMed Central PMCID: PMCPMC3705435. 10.1038/jcbfm.2013.50 23549381 PMC3705435

[42] Davies BJ, Pickard BS, Steel M, Morris RG, Lathe R. Serine proteases in rodent hippocampus. J Biol Chem. 1998;273(36):23004–11. 9722524 10.1074/jbc.273.36.23004

[43] Sappino AP, Madani R, Huarte J, Belin D, Kiss JZ, Wohlwend A, et al Extracellular proteolysis in the adult murine brain. J Clin Invest. 1993;92(2):679–85. PubMed Central PMCID: PMCPMC294901. 10.1172/JCI116637 8349806 PMC294901

[44] Lee HY, Hwang IY, Im H, Koh JY, Kim YH. Non-proteolytic neurotrophic effects of tissue plasminogen activator on cultured mouse cerebrocortical neurons. J Neurochem. 2007;101(5):1236–47. 10.1111/j.1471-4159.2007.04417.x 17498240

[45] Wind T, Hansen M, Jensen JK, Andreasen PA. The molecular basis for anti-proteolytic and non-proteolytic functions of plasminogen activator inhibitor type-1: roles of the reactive centre loop, the shutter region, the flexible joint region and the small serpin fragment. Biol Chem. 2002;383(1):21–36. 10.1515/BC.2002.003 11928815

[46] Yepes M. Tissue-type plasminogen activator is a neuroprotectant in the central nervous system. Front Cell Neurosci. 2015;9:304 PubMed Central PMCID: PMCPMC4538299. 10.3389/fncel.2015.00304 26347605 PMC4538299

[47] Leprince P, Rogister B, Delree P, Rigo JM, Andre B, Moonen G. Modulation of proteolytic activity during neuritogenesis in the PC12 nerve cell: differential control of plasminogen activator and plasminogen activator inhibitor activities by nerve growth factor and dibutyryl-cyclic AMP. J Neurochem. 1991;57(2):665–74. 1649256 10.1111/j.1471-4159.1991.tb03798.x

[48] Pang PT, Teng HK, Zaitsev E, Woo NT, Sakata K, Zhen S, et al Cleavage of proBDNF by tPA/plasmin is essential for long-term hippocampal plasticity. Science. 2004;306(5695):487–91. 10.1126/science.1100135 15486301

[49] Bernd P. The role of neurotrophins during early development. Gene Expr. 2008;14(4):241–50. 19110723 10.3727/105221608786883799 PMC6042000

[50] Crutcher KA. The role of growth factors in neuronal development and plasticity. CRC Crit Rev Clin Neurobiol. 1986;2(3):297–333. 3536312

[51] Fahnestock M, Yu G, Coughlin MD. ProNGF: a neurotrophic or an apoptotic molecule? Prog Brain Res. 2004;146:101–10. 10.1016/S0079-6123(03)46007-X 14699959

[52] Wozniak W. Brain-derived neurotrophic factor (BDNF): role in neuronal development and survival. Folia Morphol (Warsz). 1993;52(4):173–81. 8175070

[53] Edgar D. Nerve growth factors and molecules of the extracellular matrix in neuronal development. J Cell Sci Suppl. 1985;3:107–13. 3914988 10.1242/jcs.1985.supplement_3.11

[54] Stiles TL, Dickendesher TL, Gaultier A, Fernandez-Castaneda A, Mantuano E, Giger RJ, et al LDL receptor-related protein-1 is a sialic-acid-independent receptor for myelin-associated glycoprotein that functions in neurite outgrowth inhibition by MAG and CNS myelin. J Cell Sci. 2013;126(Pt 1):209–20. PubMed Central PMCID: PMCPMC3603516. 10.1242/jcs.113191 23132925 PMC3603516

[55] Strickland DK, Gonias SL, Argraves WS. Diverse roles for the LDL receptor family. Trends Endocrinol Metab. 2002;13(2):66–74. 11854021 10.1016/s1043-2760(01)00526-4

[56] Wolf BB, Lopes MB, VandenBerg SR, Gonias SL. Characterization and immunohistochemical localization of alpha 2-macroglobulin receptor (low-density lipoprotein receptor-related protein) in human brain. Am J Pathol. 1992;141(1):37–42. PubMed Central PMCID: PMCPMC1886577. 1632469 PMC1886577

[57] Bu G, Maksymovitch EA, Nerbonne JM, Schwartz AL. Expression and function of the low density lipoprotein receptor-related protein (LRP) in mammalian central neurons. J Biol Chem. 1994;269(28):18521–8. 7518435

[58] Campana WM, Li X, Dragojlovic N, Janes J, Gaultier A, Gonias SL. The low-density lipoprotein receptor-related protein is a pro-survival receptor in Schwann cells: possible implications in peripheral nerve injury. J Neurosci. 2006;26(43):11197–207. 10.1523/JNEUROSCI.2709-06.2006 17065459 PMC6674644

[59] Webb DJ, Thomas KS, Gonias SL. Plasminogen activator inhibitor 1 functions as a urokinase response modifier at the level of cell signaling and thereby promotes MCF-7 cell growth. J Cell Biol. 2001;152(4):741–52. PubMed Central PMCID: PMCPMC2195772. 11266465 10.1083/jcb.152.4.741 PMC2195772

[60] Boucher P, Gotthardt M, Li WP, Anderson RG, Herz J. LRP: role in vascular wall integrity and protection from atherosclerosis. Science. 2003;300(5617):329–32. 10.1126/science.1082095 12690199

[61] Zilberberg A, Yaniv A, Gazit A. The low density lipoprotein receptor-1, LRP1, interacts with the human frizzled-1 (HFz1) and down-regulates the canonical Wnt signaling pathway. J Biol Chem. 2004;279(17):17535–42. 10.1074/jbc.M311292200 14739301

[62] Gaultier A, Arandjelovic S, Niessen S, Overton CD, Linton MF, Fazio S, et al Regulation of tumor necrosis factor receptor-1 and the IKK-NF-kappaB pathway by LDL receptor-related protein explains the antiinflammatory activity of this receptor. Blood. 2008;111(11):5316–25. PubMed Central PMCID: PMCPMC2396725. 10.1182/blood-2007-12-127613 18369152 PMC2396725

[63] Shi Y, Mantuano E, Inoue G, Campana WM, Gonias SL. Ligand binding to LRP1 transactivates Trk receptors by a Src family kinase-dependent pathway. Sci Signal. 2009;2(68):ra18 PubMed Central PMCID: PMCPMC2696635. 10.1126/scisignal.2000188 19401592 PMC2696635

[64] Mantuano E, Mukandala G, Li X, Campana WM, Gonias SL. Molecular dissection of the human alpha2-macroglobulin subunit reveals domains with antagonistic activities in cell signaling. J Biol Chem. 2008;283(29):19904–11. PubMed Central PMCID: PMCPMC2459272. 10.1074/jbc.M801762200 18499670 PMC2459272

[65] Fuentealba RA, Liu Q, Kanekiyo T, Zhang J, Bu G. Low density lipoprotein receptor-related protein 1 promotes anti-apoptotic signaling in neurons by activating Akt survival pathway. J Biol Chem. 2009;284(49):34045–53. PubMed Central PMCID: PMCPMC2797175. 10.1074/jbc.M109.021030 19815552 PMC2797175

[66] Goto JJ, Tanzi RE. The role of the low-density lipoprotein receptor-related protein (LRP1) in Alzheimer's A beta generation: development of a cell-based model system. J Mol Neurosci. 2002;19(1–2):37–41. 10.1007/s12031-002-0008-4 12212791

[67] Prasad JM, Migliorini M, Galisteo R, Strickland DK. Generation of a Potent Low Density Lipoprotein Receptor-related Protein 1 (LRP1) Antagonist by Engineering a Stable Form of the Receptor-associated Protein (RAP) D3 Domain. J Biol Chem. 2015;290(28):17262–8. PubMed Central PMCID: PMCPMC4498065. 10.1074/jbc.M115.660084 26013822 PMC4498065

[68] Herz J, Goldstein JL, Strickland DK, Ho YK, Brown MS. 39-kDa protein modulates binding of ligands to low density lipoprotein receptor-related protein/alpha 2-macroglobulin receptor. J Biol Chem. 1991;266(31):21232–8. 1718973

[69] Hu SQ, Cui W, Mak SH, Choi CL, Hu YJ, Li G, et al Robust Neuritogenesis-Promoting Activity by Bis(heptyl)-Cognitin Through the Activation of alpha7-Nicotinic Acetylcholine Receptor/ERK Pathway. CNS Neurosci Ther. 2015;21(6):520–9. 10.1111/cns.12401 25917415 PMC6495446

[70] Vizard TN, Newton M, Howard L, Wyatt S, Davies AM. ERK signaling mediates CaSR-promoted axon growth. Neurosci Lett. 2015;603:77–83. PubMed Central PMCID: PMCPMC4552171. 10.1016/j.neulet.2015.07.019 26200251 PMC4552171

